# Overcoming anatomical barriers in pediatric kidney transplantation

**DOI:** 10.1007/s00467-025-07077-6

**Published:** 2025-12-02

**Authors:** Fabian Eibensteiner, Thomas Mueller-Sacherer, Siegfried Waldegger, Stefan Scheidl, Christoph Aufricht, Azadeh Hojreh, Sabine Wipper, Stefan Schneeberger, Katrin Kienzl-Wagner

**Affiliations:** 1 Division of Pediatric Nephrology and Gastroenterology, Department of Pediatrics and Adolescent Medicine, Comprehensive Center for Pediatrics, Medical University of Vienna, Vienna, Austria; 2 Department of Pediatrics, Medical University of Innsbruck, Innsbruck, Austria; 3 Department of Visceral, Transplant and Thoracic Surgery, Medical University of Innsbruck, Innsbruck, Austria; 4 Department of Biomedical Imaging and Image Guided Therapy, Medical University of Vienna, Vienna, Austria; 5 Department of Vascular Surgery, Medical University of Innsbruck, Innsbruck, Austria

**Keywords:** Kidney transplantation, Live donor kidney transplant, Vascular access, Inferior vena cava occlusion, Azygos continuation, Gore-Tex graft, Size-mismatch

## Abstract

**Background:**

Pediatric kidney transplantation in patients with inherited disorders and associated congenital anomalies constitutes a true challenge, especially in low weight recipients (< 15–20 kg). Potential barriers for a successful transplant involve size-mismatch between donor organ and recipient as well as identification of suitable vascular anastomotic sites in case of vascular anomalies.

**Case - diagnosis/treatment:**

We present a case of a 5-year-old girl with azygos continuation of the inferior vena cava (IVC), a condition generally precluding transplantation of an adult-sized kidney as adequate venous outflow from the transplant cannot be achieved. By interposing a prosthetic vascular graft, the donor renal vein was anastomosed to the suprahepatic IVC, thereby enabling transplantation of a live donor kidney to the pediatric recipient with the technical success being reflected in immediate graft function.

**Conclusion:**

Anatomical barriers such as azygos continuation of the IVC do not preclude kidney transplantation from a size-mismatched adult live donor a priori but demand a patient-tailored innovative surgical approach and a committed multidisciplinary perioperative management to potentially overcome this kind of vascular anomaly.

## Case report

A 5-year-old girl presented with an indication for kidney transplantation. The girl was born preterm (35 + 4 weeks, low birth-weight of 1650 g) with a cleft lip and cleft palate. She had two distinct chromosomal deletion syndromes (6p25.1p23 and TFAP2A [transcription factor activating enhancer binding protein 2 alpha], branchio-oculo-facial syndrome). Following repeat cardiac surgery during infancy due to ventricular and atrial septal defects as well as peripheral pulmonary stenosis, the patient developed secondary ischemic nephropathy. At presentation for pre-transplant work-up, the girl was severely growth-retarded (17 kg [23rd percentile], 91 cm [<  1 st percentile]), displayed psychomotor retardation, was fed mainly via a percutaneous endoscopic gastrostomy, had tracheostomy for home respiration with continuous positive airway pressure (CPAP) when sleeping, and was on peritoneal dialysis. Decline of kidney function leading to fluid overload but also increased abdominal pressure during peritoneal dialysis led to an increase in CPAP use and tracheostomy-related infections.

## Transplant options in low weight pediatric recipients

Generally, kidney transplantation from a living donor, in contrast to deceased donation, leads to better graft survival outcomes in children with kidney failure, although this gap has been narrowing in recent years [1–4]. More precisely, transplantation of an adult-sized kidney from a living donor is the best choice for children with kidney failure both in terms of short- and long-term allograft survival and function [11]. A recent analysis of a large European pediatric cohort highlighted that a significant proportion of small children receive a kidney transplantation at very low body weights, with equally good outcomes five years after transplantation [12]. Nevertheless, kidney transplantation in pediatric recipients with a body weight of < 15–20 kg implies some technical challenges. Transplantation of an adult-sized kidney to a small recipient is complicated by the size mismatch between the donor organ and the recipient. Both the graft and recipient vessel size as well as hemodynamic considerations necessitate graft vascular anastomoses directly to the aorta and IVC to ensure adequate arterial in- and venous outflow from the allograft. On the other hand, transplantation of small pediatric donor kidneys to small recipients requires advanced microsurgical skills as both donor and recipient vessels are extremely small in diameter, therefore increasing the risk of graft vascular thrombosis [13].

Live donor kidney transplantation not only results in superior transplant outcomes [1–4], but also has logistical advantages over deceased donor transplantation. Live donor kidney transplantation is an elective procedure that can be planned. This is particularly important in complex patients who require optimal perioperative care from pediatric anesthetists, specialists in pediatric intensive care medicine, pediatric nephrologists and pediatric transplant surgeons. For these reasons, live donor kidney transplantation was considered the optimal strategy for the patient described herein who has a rare inherited disorder and a complicated medical history.

However, advancing live donor kidney transplantation from her grandmother, pre- transplant work-up of the girl revealed azygos continuation of the inferior vena cava (IVC) [14]. Azygos continuation of the IVC is a rare congenital anomaly that results from aberrant development of the inferior vena cava during embryogenesis [15]. It has a prevalence of 0.6% and is characterized by the absence of the hepatic segment of the IVC, i.e. the IVC is interrupted below the hepatic veins. Venous return is restored by the dilated azygos and hemiazygos veins draining into the superior vena cava. Azygos continuation of the IVC is classically associated with polysplenia, cardiovascular malformations and situs anomalies. Without adequate collateral pathways it can result in deep vein thrombosis and/or venous insufficiency [16]. Generally, occlusion of the IVC (either through complete thrombosis or resulting from congenital venous anomaly) precludes a live donor kidney transplant in low weight recipients because adequate renal venous outflow from the adult-sized graft cannot be achieved.

## What are the options in this specific case with atresia of the IVC?

### Consider a small pediatric donor kidney for transplantation

Kidney transplantation from a small pediatric donor (< 15-kg body weight) is an alternative with better size-matching. Still, for a long time the use of small pediatric donor kidneys has been associated with an increased risk for graft loss in the early postoperative period, presumably due to higher rates of vascular complications. However, only recently de Santis et al. have shown that regarding patient and graft survival the use of small pediatric donor kidneys for pediatric recipients is equally successful as transplantation of bigger pediatric donor kidneys or adult living donor kidneys when the procedure is performed in a specialized center [17].

In the presence of a congenital anomaly of the systemic venous return, as in this case, an alternative venous anastomosis site needs to be identified. Several target veins for renal vein anastomosis in this context have been described: an ascending lumbar vein, the azygos vein, the recipient’s renal vein, the gonadal vein, the portal vein, the splenic vein, the inferior mesenteric vein, or the superior mesenteric vein. Additionally, a venous interposition graft may be required to bridge the distance between donor organ and target vein [18, 19]. In the case presented herein, however, the pre-transplant MRI did not reveal a promising target vein with sufficient diameter for anastomosis of a potential pediatric donor organ. Therefore, kidney transplantation from a deceased donor in this scenario would necessitate laparotomy with exploration of the vascular status immediately preceding the transplant. Only in case a suitable target vessel was identified could the transplant procedure be continued and performed.

### Proceed with live donor kidney transplantation

As mentioned above, one of the major advantages of living donor kidney transplantation over deceased donor transplantation is that it can be scheduled and therefore ensures optimal perioperative care from a dedicated multidisciplinary pediatric team. Building on the report of Verghese et al. [19], we therefore aimed at realizing a live donor transplant in our patient. Verghese and colleagues successfully implanted an adult-sized kidney graft in two pediatric recipients with complete occlusion of the IVC (9.8 kg and 14 kg body weight, respectively) by anastomosing the donor renal vein to the right hepatic vein/IVC junction. The kidney graft was positioned posterior to the right liver lobe [19].

## Case continued — the transplant

**Fig. 1:**
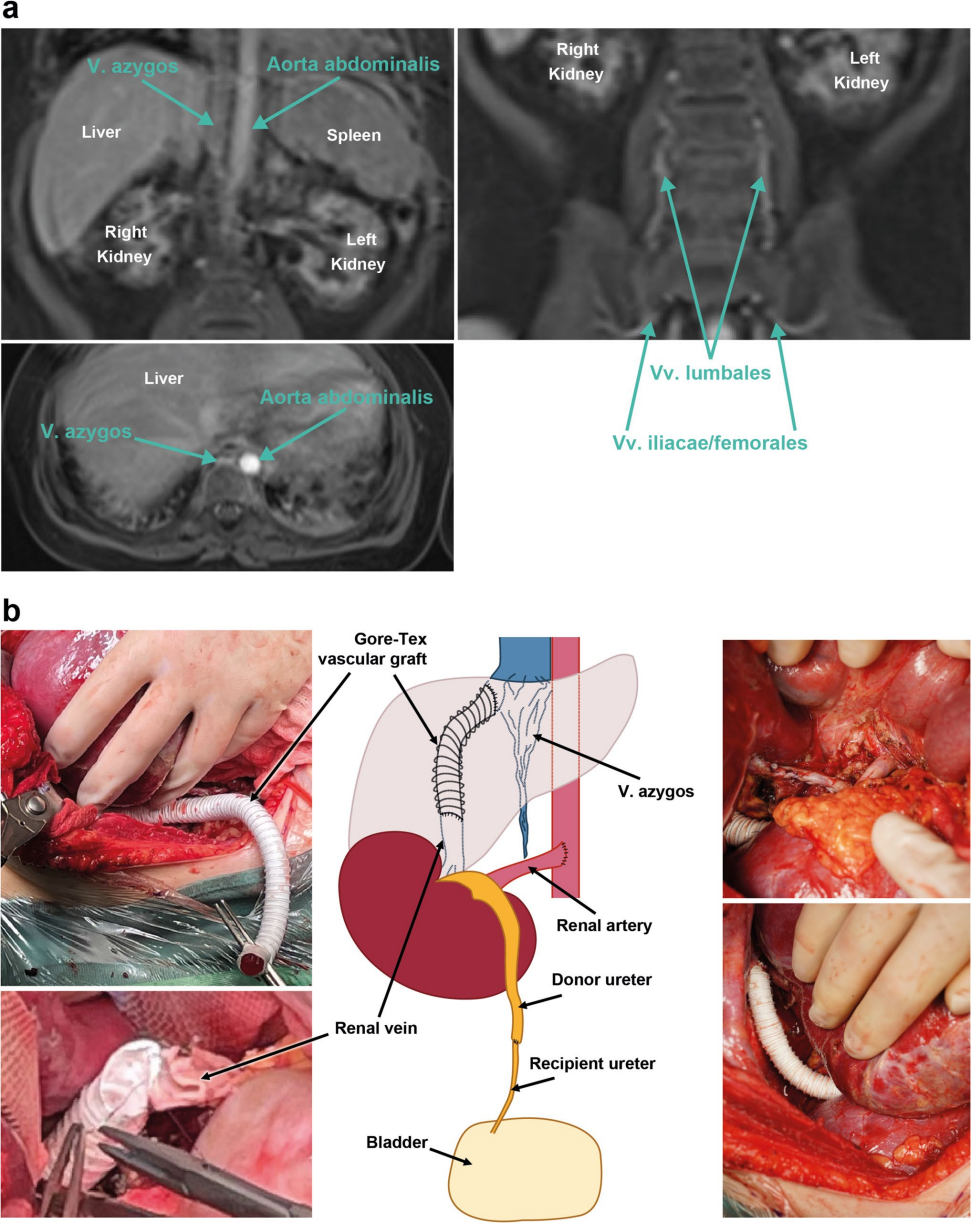
Panel (**a**) Magnetic Resonance Imaging (MRI) with contrast media (venous phase) showing azygos continuation of the inferior vena cava. The azygos vein is formed from the renal and lumbal veins at the level of L2. Panel (**b**) Diagram and intraoperative photographs illustrating the sites of arterial, venous, and ureteral anastomoses, including interposition of a Gore-Tex graft (10 mm) E/S to the suprahepatic IVC and E/E to the orifice of the donor renal vein

Live donor kidney transplantation was performed using the left kidney from the girl’s 58-year-old grandmother (HLA mismatch A/B/DR 1–1-0, donor/recipient serostatus for CMV −/− and EBV +/−, donor height 164 cm, donor weight 90 kg). Following laparotomy via a right-sided L-shaped incision, the abdomen was meticulously inspected for alternative venous anastomotic sites not evident from pre-transplant imaging, yet without success. The infrahepatic IVC was identified as an atretic vessel with no visible lumen. Hence the right lobe of the liver was completely mobilized to expose the subdiaphragmatic IVC for venous anastomosis as described by Verghese et al. For reasons of space, right-sided recipient nephrectomy was necessary. To reduce the bridging distance between the graft renal vein and the right hepatic vein/inferior vena cava junction, the donor kidney (11 cm × 4.6 cm × 5.6 cm) was placed orthotopically into the right hemiabdomen. The donor renal artery was anastomosed end-to-side to the abdominal aorta (Polydioxanone [PDS] 6/0 running suture). The donor renal vein was anastomosed to the suprahepatic IVC. To achieve adequate vessel length a ringed Gore-Tex graft (length 9 cm, diameter 10 mm corresponding to the diameter of the donor renal vein) was interposed as a vascular conduit attaching the prosthetic vascular graft end-to-side to the suprahepatic IVC and end-to-end to the orifice of the donor renal vein (Polypropylene [prolene] 6/0 running sutures, Fig. 1). The urinary tract was reconstructed via an end-to-end ureteroureterostomy to the right native ureter (PDS 7/0 interrupted suture) involving placement of a double-J catheter (6 Fr, 12 cm). The immunosuppressive regimen consisted of basiliximab and corticosteroids (methylprednisolone) for induction, and tacrolimus, mycophenlate mofetil, and corticosteroids (prednisolone) for maintenance therapy. Corticosteroids were reduced weekly and kept at a dosage of 4 mg/m^2^/day.

The kidney graft functioned immediately with appropriate diuresis and a 24-h creatinine decline to 1 mg/dl. Postoperative anticoagulation included heparin for the first 14 days, dual platelet anticoagulation (acetylsalicylic acid [aspirin] and clopidogrel) was added on day 7. Apart from an abdominal wall hematoma that required surgical evacuation the postoperative course was uneventful. The girl was discharged with an estimated glomerular filtration rate (eGFR) of 127 ml/min/1.73 m^2^ (0.39 mg/dl creatinine) 4 weeks post-transplant and continued to have excellent graft function. Within weeks the girl’s motor skills and speech improved substantially, as observed by her caregivers, physicians, and psychologists who had performed previous neurocognitive evaluations.

Neurocognitive performance of children with kidney failure in comparison to their healthy siblings, is significantly worse [9]. Children with kidney transplantation and other comorbid conditions score significantly lower in full scale intelligence quotient (IQ) tests and verbal ability assessments [20]. The time period of impaired kidney function also seems to play a role in neurocognitive status, as fewer months on dialysis and younger age at transplant are associated with better neurodevelopmental and neurocognitive outcomes [9, 21]. Though convincing, published data on neurocognitive performance in children with kidney failure are limited to small sample sizes, and the impact of chronic immunosuppressive therapy on neurocognitive outcomes is still poorly understood [10]. However, in our patient, the benefits of kidney transplantation were striking. Before transplantation the girl communicated with rudimentary sign language only, whereas briefly after the transplant she developed first elements of speech with a speaking valve for her tracheostomy. In addition, progressive improvements of fine motor skills were observed. And most importantly, the need for intermittent CPAP ventilation substantially decreased (due to decreased abdominal pressure without the need for peritoneal dialysis and improved fluid balance) resulting in enhanced quality of life for the patient and her family. Three months following kidney transplantation, however, the girl unfortunately died at home due to sudden cardiac death. A postmortem was not conducted.

Children with dialysis obviously experience lower health-related quality of life. A recent longitudinal multi-center study on 377 children with chronic kidney disease of all stages demonstrated that improvement in health-related quality of life over time is, indeed, driven by the transition from dialysis to transplantation [6]. For very young patients as well as developmentally impaired patients, proxy reports by the caregivers provide additional relevant information. As a recent study by Alaskar et al. points out, quality of life is rated significantly higher following transplantation compared to dialysis both by the pediatric patients and their parents/caregivers [5]. These data mirror the overarching desire for normality in daily life that both patients with kidney failure and their families long for [22].

Nevertheless, as other retrospective studies highlight, beyond improvements on psychosocial well-being favorable graft outcomes of kidney transplantation in children with severe motor and intellectual disabilities are achievable, but require careful perioperative management and continued medical care by a dedicated interdisciplinary team (Table 1) [23–26].

**Table 1:** Key management points and their respective level of evidence (as defined per the Oxford centre for evidence-based medicine levels of evidence)

Key management point	Level of evidence
Live donor kidney transplantation is superior to deceased donor kidney transplantation with respect to long-term patient and allograft survival and hence is the preferred treatment option for pediatric patients with kidney failure [1–4]	2b (Retrospective cohort study)
Size mismatch between an adult-sized donor organ and the recipient may preclude live donor kidney transplantation in patients with < 10 kg body weight	5 (Expert opinion)
Transplantation of an adult-sized donor organ in low weight pediatric recipients necessitates patency of the abdominal aorta and inferior vena cava for anastomoses of graft renal artery and vein. Therefore, abdominal imaging is mandatory during pre-transplant work-up to identify or rule out vascular anomalies [18, 19]	4 (Case-series)
Rare inherited disorders and associated anomalies of the vascular system or the genitourinary tract constitute a true surgical challenge in pediatric kidney transplantation	5 (Expert opinion)
Congenital or acquired vascular anomalies do not preclude kidney transplantation but require a patient-tailored surgical approach, meticulous pre-transplant work-up and perioperative patient management by a dedicated multidisciplinary team	5 (Expert opinion)
A successful transplant is not only defined by prolongation of life but equally important by enhanced health-related quality of life [5–7] and neurocognitive improvement [8–10]	2b (Retrospective cohort study) and 4 (Case-series)

## Discussion

Consistent with Verghese et al. [19], we have demonstrated that in case of azygos continuation of the IVC transplantation of an adult-sized kidney allograft into a pediatric recipient is feasible by anastomosing the donor renal vein to the right hepatic vein/suprahepatic IVC. Since a suitable venous allograft was not available to lengthen the donor renal vein, a prosthetic vascular graft was interposed instead. To prevent wall collapse of the prosthesis by extrinsic compression through the liver a ringed Gore-Tex graft was used. This was critically important in this case, since the liver was resting on the vein and its weight would have compressed the interpositioned graft. Although in the context of live donor liver transplantation synthetic grafts are often used for middle hepatic vein reconstruction [27] there are only sparse reports on renal vein reconstruction with polytetrafluoroethylene (PTFE) vascular grafts in kidney transplantation [28–30]. In these cases, the PTFE grafts were used to lengthen donor renal veins that were either too short or had been damaged, hence, the prosthetic grafts were rather short (i.e., 2.5 cm and 4 cm [29, 30]). In contrast, due to azygos continuation of the IVC, the PTFE graft in our patient needed to be quite long (9 cm, diameter 10 mm) to bridge the distance between the donor kidney and the suprahepatic IVC. Reported patency rates in these small case series ranged between 0.5 and 10 years, limited by published observation time [28–30].

Albeit a venous allograft would have been the preferred option for vascular reconstruction, lack of availability of such a venous allograft necessitated use of a synthetic graft. According to the published literature on the use of PTFE grafts for middle hepatic vein reconstruction in living donor liver transplantation, the incidence of PTFE-related complications such as infection is low (up to 4.7%) [31, 32] and patency rates are comparable to cryopreserved iliac veins (6-month patency 76.6% PTFE vs. 75.3% cryopreserved iliac vein) [33].

## Conclusion

Pediatric kidney transplantation is unique in a way, that rare inherited disorders and associated congenital anomalies of the genitourinary tract or the vascular system may constitute a true surgical challenge. Here, we report a successful kidney transplantation regarding technical feasibility and short-term graft function in a child with azygos continuation of the IVC with interposition of a prosthetic vascular graft between the donor renal vein and the suprahepatic IVC to achieve adequate venous outflow from the kidney graft. However, interpretation of middle- to long-term outcome is limited due to the sudden death of the patient three months post-transplant. In conclusion, congenital or acquired vascular anomalies in patients with kidney failure do not preclude kidney transplantation a priori but require a patient-tailored creative surgical approach. And it is the combination of both surgical technique and thoughtful preoperative planning that accounts for a successful transplant. Apart from the technical success and graft survival, outcome after pediatric kidney transplantation is also defined by improvement in neurocognitive development, motor skills, and health-related quality of life for both the pediatric patients and their parents/caregivers.

## Data Availability

All data are included in the manuscript.
